# Data that effectively demonstrate the benefits of a 3D CAPPI algorithm

**DOI:** 10.1016/j.dib.2019.104116

**Published:** 2019-06-11

**Authors:** Yura Kim, Masayuki Maki, Dong-In Lee

**Affiliations:** a Research and Education Center for Natural Hazards, Kagoshima University, Japan; b Department of Environmental Atmospheric Sciences, Pukyong National University, Busan, South Korea

**Keywords:** High-resolution, Three-dimensional, X-band polarimetric radar, Convective precipitation, Initial stage

## Abstract

The data presented in this article are related to the research article entitled “Three-dimensional analysis of the initial stage of convective precipitation using an operational X-band polarimetric radar network” [1]. The data presented were obtained using a three-dimensional constant-altitude plan-position-indicator (3D CAPPI), which was generated by a new method proposed by [1]. The data used to create the 3D CAPPI were derived from two X-band polarimetric radar installations in the Kanto region of Japan, Ebina (139.39°E, 35.40°N), and Shin-yokohama (139.60°E, 35.51°N). These data are superior to operational radar data in terms of their temporal and spatial resolution. These high resolution data can indicate a rapidly developing storm, such as localized precipitation. It is particularly important to understand the early stages of storms in terms of numerical and short-term models. These data show the time of appearance, life cycle, and evolution of each cell that constitutes a storm in three-dimensional detail.

**Table:** Specifications table

Subject area	*Atmospheric Research*
More specific subject area	*Meteorology*
Type of data	*Animation files*
How data were acquired	*X-band polarimetric radar observations*
Data format	*Raw, filtered, and analyzed*
Experimental factors	*The data were collected at* 5 min *intervals*
Experimental features	*High-spatiotemporal-resolution volumetric data modified using a new algorithm.*
Data source location	*Kanto region of Japan: Ebina (139.39°E, 35.40°N) and Shin-yokohama (139.60°E, 35.51°N).*
Data accessibility	*The data are presented with this article as a supplementary animation file*
Related research article	*Y. Kim, M. Maki, D.-I. Lee, J.-H. Jeong, C.-H. You, Three-dimensional analysis of the initial stage of convective precipitation using an operational X-band polarimetric radar network. Atmos. Res. 225, 2019, 45–57*[1].

**Table:** 

**Value of the data** • The data presented here are considered to be superior to existing radar data in terms of their temporal and spatial resolution. The data demonstrate the overall storm structure and temporal evolution during its initial stage. • The data provide several polarimetric parameters. Using these polarimetric parameters, other researchers can study the microphysical processes related to the evolution of storms [2], [3], [4], [5]. • This data can assist in making accurate forecasts by improving the initial conditions in numerical and short-term models [6]. • The research data provide users with more detailed information for targets observed by radar. The algorithm generating these data can be applied to various targets that can be observed by radar [7], [8].

## 1 Data

**Fig. 1:**
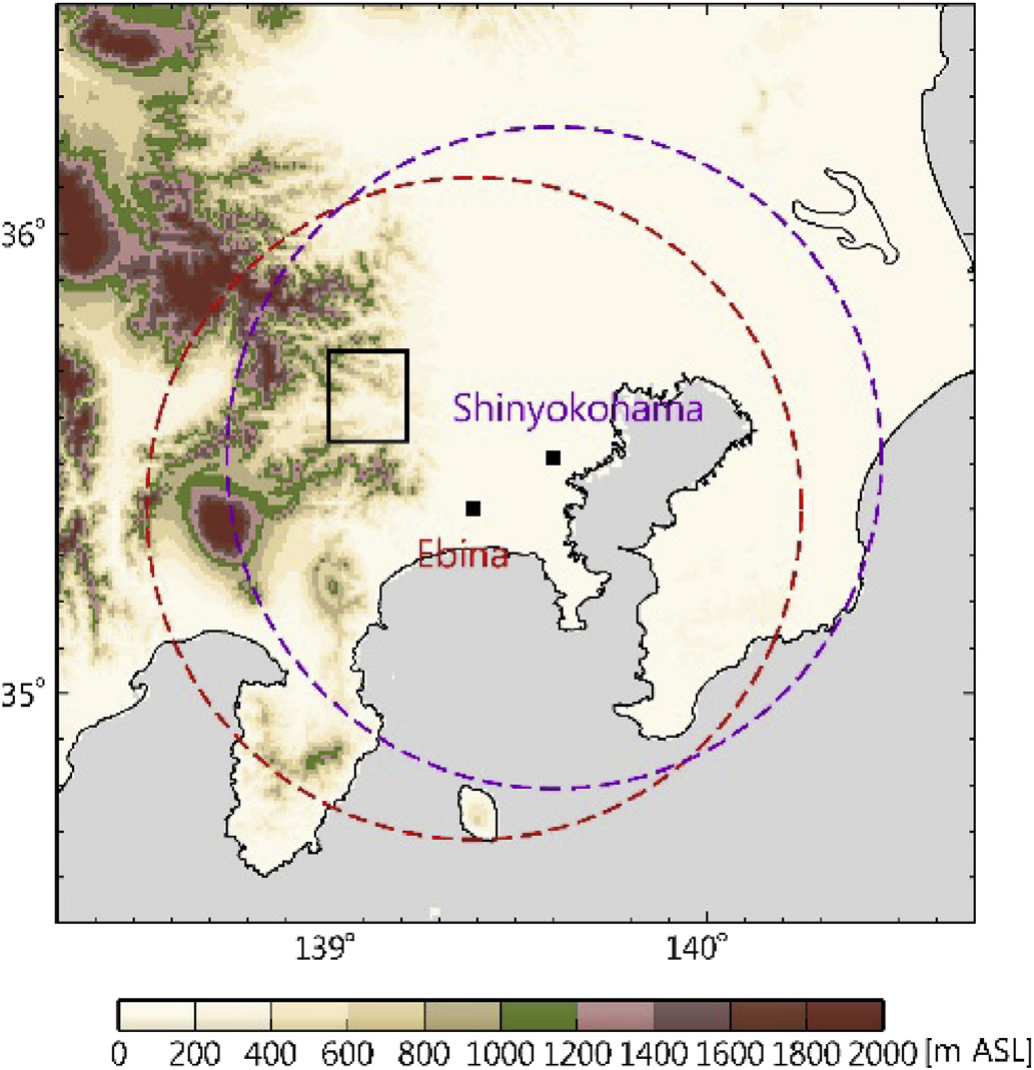
Radar sites are indicated by black squares (■). In the Kanto region of Japan, the Ebina radar is located at 139.39°E, 35.40°N and the Shin-yokohama radar is located at 139.60°E, 35.51°N. The ranges of the EBN and SYK radars are shown by the dashed red and violet lines, respectively (80 km range). The target area is enclosed by the black rectangle.

**Fig. 2:**
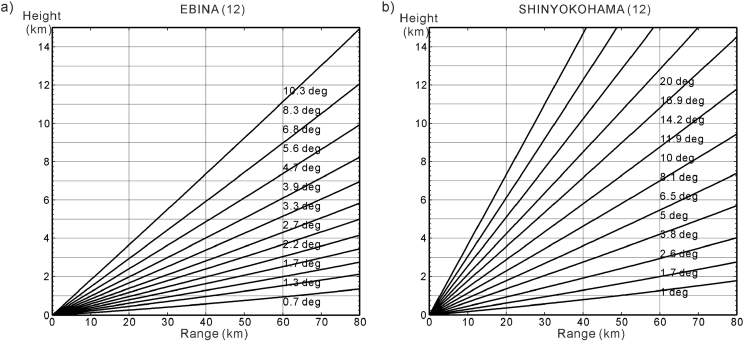
Beam-height cross sections of the (a) EBN radar and (b) SYK radar [1].

The data presented here were observed by two radar installations in the Kanto area on 19 July 2012. The observation target was a rapidly developing storm that evolved over a short period of time and was observed on radar from 1210 LST to 1249 LST. The specifications of the two X-band polarimetric radars are shown in Table 1, while Fig. 1 shows the location of the radar installations and the area of the observation target. The beam-height cross sections in Fig. 2 [1] shows elevation angles for each radar.

**Table 1:** Specifications of the radar installations.

Radar site	Ebina	Shin-yokohama
Type	X-band polarimetric radar	X-band polarimetric radar
Observation range	80 km	80 km
Volume scan interval	5 min	5 min
Operation agency	NIED	MLIT
Location	139.39°E, 35.40°N	139.60°E, 35.51°N
Number of elevation angles	12 (0.7–10.3°)	12 (1.0–20.0°)
Data resolution	100 m (range), 1.0° (azimuth)	150 m (range), 1.2° (azimuth)
Beam width	1.3°	1.05°
Frequency	9.375 GHz	9.7–9.8 GHz
PRF	≤1800 Hz	1440–1800 Hz

NIED: National Research Institute for Earth Science and Disaster Prevention, MLIT: Ministry of Land, Infrastructure, Transport and Tourism, PRF: Pulse Repetition Frequency.

## 2 Experimental design, materials, and methods

### 2.1 High spatio-temporal resolution data

The data presented here were derived from observational radar data, with the application of temporal and spatial interpolation [1]. The high-resolution data were created at 30-s intervals in time (Animation 1 of Appendix A) and 0.1° intervals in space (Animation 2 of Appendix A).

While operational radar data (left image in Animation 1) show the temporal evolution of a storm at 5-min intervals, high-temporal-resolution data (right image in Animation 1) can provide more detail, as demonstrated in the time evolution at 30-s intervals. Because the advection vector updates by calculating for a period of volume scan interval [1], a storm in the high-temporal-resolution data do not move smoothly (such as a jumping downward movement shown in the right image in Animation 1). The left image of Animation 2 shows observational radar data with 12 elevation angles before elevation interpolation from 1235 LST to 1239 LST; the right image shows 0.1° high-spatial-resolution radar data with 97 elevation angles at 1238:30 LST.

### 2.2 Implementation of three-dimensional storm information

Using the high spatio-temporal resolution data, a three-dimensional constant-altitude plan-position-indicator (3D CAPPI) was created at a resolution of 250 × 250 × 250 m. The 3D CAPPI described the evolution of the storm at various angles. Investigating the evolution of a storm from various angles is useful for understanding storm structure, development, and movement (Animation 3–6 of Appendix A).
